# Ecologic study of influenza vaccination uptake and COVID-19 death rate in New York City

**DOI:** 10.1186/s12889-022-13515-z

**Published:** 2022-06-01

**Authors:** Ashley Moreland, Christina Gillezeau, Adriana Eugene, Naomi Alpert, Emanuela Taioli

**Affiliations:** Institute for Translational Epidemiology, Icahn School of Medicine at Mount Sinai, New York, USA

**Keywords:** Influenza vaccination, COVID-19, Socioeconomic factors

## Abstract

**Background:**

The aim of this ecological study was to assess the area-level relationship between cumulative death rate for COVID-19 and historic influenza vaccination uptake in the New York City population.

**Methods:**

Predictors of COVID-19 death included self-reported influenza vaccination in 2017, as well as four CDC-defined risk factors of severe COVID-19 infection available at the ecological level, which were diabetes, asthma, BMI 30–100 (^2 kg/m2^) and hypertension, in addition to race and age (65 + years).

**Results:**

After adjusting for potential confounders, for every one-unit increase in influenza vaccination uptake for each zip code area, the rate of COVID-19 deaths decreased by 5.17 per 100,000 residents (*p* < 0.0001).

**Conclusions:**

Zip codes with a higher prevalence of influenza vaccination had lower rates of COVID-19 mortality, inciting the need to further explore the relationship between influenza vaccination uptake and COVID-19 mortality at the individual level.

## Background

New York City was an early and prominent epicenter of the coronavirus (COVID-19) pandemic in the United States, with over 360,000 cases and 24,000 deaths [1] as of December 2, 2020. Even though COVID-19 vaccinations are widely available, demand has tapered [2], and many Americans are still hesitant to get vaccinated [3]. As such, it is necessary to understand why some people had worse or better outcomes by assessing similarities in how people respond to different respiratory viruses.

Influenza vaccination has been postulated to mitigate COVID-19 severity [4], particularly in elderly populations [5, 6]. However, there are racial and socio-economic factors associated with both COVID-19 severity [7] and the likelihood of getting an influenza vaccine [7] that could act as confounders. Although previous studies have been conducted in Italy [5, 6] and two non-peer reviewed articles in the United States and Brazil, none include these possible confounders. For the first time, we assessed the area-level relationship between cumulative death rate for COVID-19 and historic influenza vaccination uptake in New York City and adjusted for possible confounders, including racial factors and COVID-19 severity, as defined by the Centers for Disease Control and Prevention (CDC) [8].

## Methods

An ecological analysis was conducted at the modified zip code tabulation area (MODZCTA) level; there are 178 MODZCTAs in New York City. Cumulative COVID-19 death rates through December 2, 2020 were extracted by MODZCTA from the New York City (NYC) Department of Health (DOH) Coronavirus repository. Age-adjusted prevalence (%) of self-reported influenza vaccination in the previous year, diabetes, asthma, BMI ≥ 30 kg/m^2^, and hypertension by United Hospital Fund (UHF) neighborhood were sourced from the 2017 Community Health Survey (CHS) via the Department of Health and Mental Hygiene’s EpiQuery tool [9]. Inclusion of diabetes, asthma, BMI ≥ 30 kg/m^2^, and hypertension was based on CDC-defined risk factors of severe COVID-19 infection [8]. Other covariates of interest, including the percent of residents who were white, Hispanic, and ≥ 65 years old in each ZCTA, were extracted from the American Community Survey (ACS) 2018 5-year estimates [10]. Data for CHS and ACS indicators were converted to MODZCTA using crosswalks provided by the NYC DOH [11, 12]. This study is exempt from IRB approval as data used is de-identified and publicly available. We conducted a multivariable linear regression analysis to assess the MODZCTA level association of COVID-19 death rates with prevalence of influenza vaccination in 2017, adjusting for possible confounders. All methods were carried out in accordance with relevant guidelines and regulations' or the 'Declaration of Helsinki'.

## Results

Overall, in NYC in 2017, 44% of residents reported receiving an influenza vaccine in the previous year, 13% reported having asthma, 25% reported having a BMI ≥ 30 kg/m^2^, and 28% reported having hypertension. The overall population of NYC was 43% white, 29% Hispanic, and 15% 65 years or older (Table 1).

**Table 1:** Multiple linear relationship between COVID-19 death rate and influenza vaccination prevalence in NYC residents

Variable (%)	COVID-19 Cumulative Death Rate per 100,000^a^						
	Median (IQR)	Q1	Q3	Coefficients	*p*	95% Confidence	
				B		Lower	Upper
Influenza Vaccination Prevalence^b^	44 (7.2)	40.7	47.9	-5.17	< 0.0001	-7.4	-2.93
Diabetes Prevalence^b^	11.2 (6.0)	9	15	3.86	0.1736	-1.72	9.44
Asthma Prevalence^b^	13.4 (7.2)	10	17.2	1.28	0.4169	-1.82	4.38
BMI ≥ 30 kg/m^2^ Prevalence^b^	23.3 (11.3)	19.1	30.4	0.89	0.6007	-2.45	4.22
Hypertension Prevalence^b^	28.2 (9.0)	23.5	32.5	-2.05	0.3635	-6.5	2 .39
Proportion White Residents^c^	46.4 (45.2)	22.62	67.87	-1.71	< 0.0001	-2.42	-1
Proportion Hispanic Residents^c^	18.9 (26.2)	10.91	37.12	1.58	0.0002	0.76	2.4
Proportion Residents ≥ 65 Years^c^	13.6 (6.0)	11.11	17.07	11.98	< 0.0001	9.1	14.86

^a^New York City Department of Health. NYC Coronavirus disease 2019 (COVID-19) data. https://github.com/nychealth/coronavirus-data. Accessed December 2, 2020

^b^Department of Health and Mental Hygiene NYCDoHaMH EpiQuery. https://nyc.gov/health/epiquery. Accessed November 12, 2020

^c^Bureau USC. American Community Survey. 2017 American Community Survey 5-Year estimates. https://data.census.gov/cedsci/. Accessed April 28, 2020

**Fig. 1:**
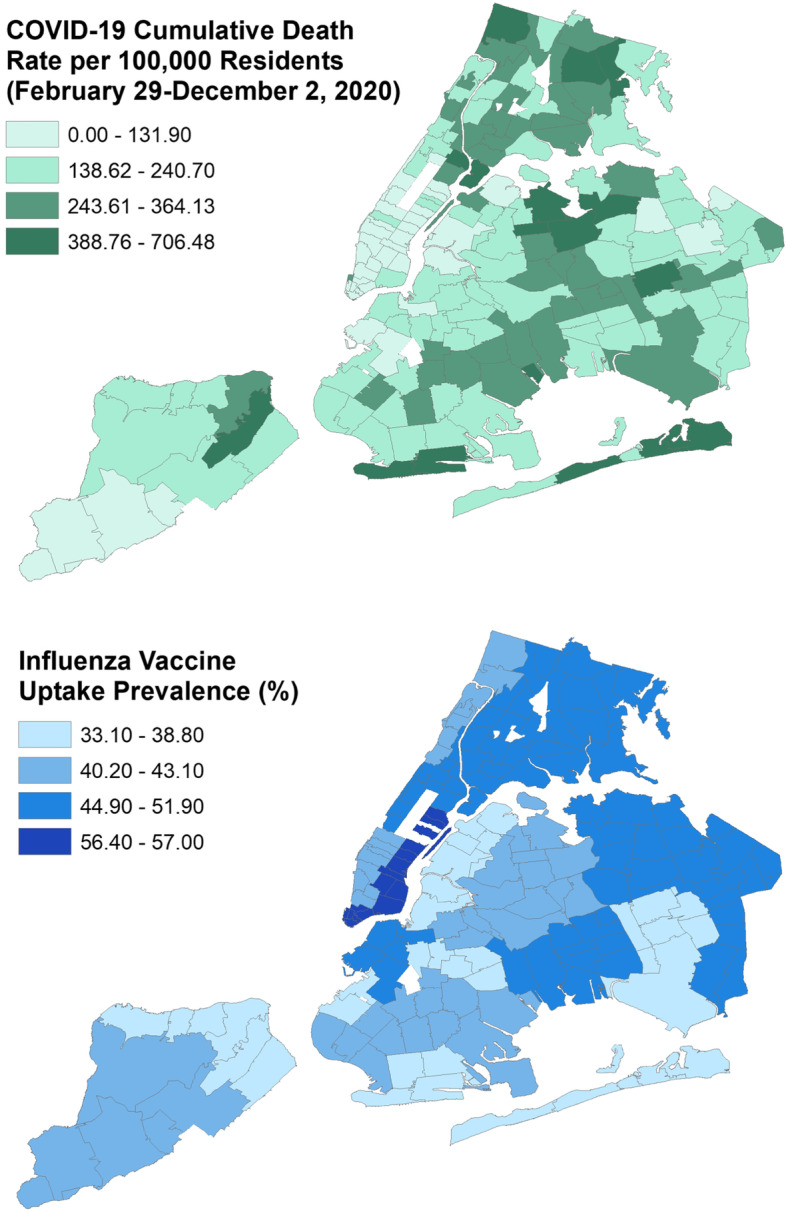
Distribution of the COVID-19 cumulative death rate per 100,000 residents February 29-December 2, 2020 (top) and age-adjusted prevalence (%) of self-reported influenza vaccination (bottom) from UHF 2017 CHS across 178 NYC Modified Zip Code Tabulation Areas

After adjusting for potential confounders, predictors accounted for 49% of the variability in the COVID-19 death rate (*p* < 0.0001), and for every one-unit increase in influenza vaccination uptake for each zip code area, the rate of COVID-19 deaths decreased by 5.17 per 100,000 residents (*p* < 0.0001) (Fig. 1). The proportion of white residents (B_adj_ = -1.710, *p* < 0.0001) was also significantly inversely associated with mortality. Older age (B_adj_ = 11.980, *p* < 0.0001) and the proportion of Hispanic residents were positively associated with COVID-19 mortality (B_adj_ = 1.580, *p* = 0.0002).

## Discussion

We show here for the first time, to our knowledge, that New York City neighborhoods with a higher prevalence of influenza vaccination had lower rates of COVID-19 mortality, even after adjustment for racial and ethnic makeup, and distribution of age and health risk factors for severe COVID-19. While the association with risk factors for severe COVID-19 was tempered after adjustment, our results still reveal some racial/ethnic disparities in the COVID-19 death rate, which may be indicative of reduced health care accessibility among minority populations [13] and increased risk for community spread [14]. However, a major limitation of this analysis is that the neighborhood-level study design hinders our ability to come to individual-level conclusions. Additionally, influenza vaccination uptake may indirectly indicate better access to healthcare in general, which may contribute to reduced COVID-19 severity. Further, it is also possible that people who get an influenza vaccine may have better health knowledge, and may have been more likely to engage in health behaviors that would reduce the risk of infection, such as masking and social distancing. Influenza vaccine data are from 2017, as the CHS does not report more recent data, and the duration of the heterologous or non-specific effects of vaccines is unknown.

This analysis is the first to consider these confounders in a US based database and contribute this information to the current literature on COVID-19. These findings suggest that influenza vaccination may have contributed to lower COVID-19 mortality. Further examining this relationship may help elucidate our understanding of population wide prevention and mitigation measures for other respiratory viruses. We suggest that future research should explore the relationship between influenza vaccination uptake and COVID-19 mortality at the individual level.

## Conclusion

In light of this study, influenza vaccination should be actively promoted, in addition to the COVID-19 vaccine, as it may bolster public health, particularly for those not yet eligible for a COVID-19 vaccine.

## Data Availability

The data that support the findings of this study are openly.

## Abbreviations

COVID-19: Coronavirus disease of 2019
CDC: Centers for Disease Control and Prevention
MODZCTA: Modified Zip Code Tabulation Area
NYC: New York City
DOH: Department of Health
UHF: United Hospital Fund
CHS: Community Health Survey
ACS: American Community Survey
